# Loss of 5-hydroxymethylcytosine as a Poor Prognostic Factor for Primary Testicular Diffuse Large B-cell Lymphoma

**DOI:** 10.7150/ijms.65517

**Published:** 2022-01-01

**Authors:** Ye Shen, Lihong Wang, Jinping Ou, Bingjie Wang, Xinan Cen

**Affiliations:** Department of Hematology, Peking University First Hospital, No. 8, Xishiku Street, Xicheng District, Beijing, China, 100034.

**Keywords:** 5-hydroxymethylcytosine, prognostic factor, primary testicular diffuse large B-cell lymphoma, immunohistochemistry.

## Abstract

**Background**: 5-Hydroxymethylcytosine (5-hmC), a stable epigenetic marker, is closely related to tumor staging, recurrence and survival, but the prognostic value of 5-hmC in primary testicular diffuse large B-cell lymphoma (PT-DLBCL) remains unclear. This study aimed to investigate the 5-hmC expression in PT-DLBCL and evaluate its prognostic value.

**Methods:** A total of 34 patients with PT-DLBCL treated in the Department of Hematology from August 2000 to August 2020 were included in this study. The expression of 5-hmC in PT-DLBCL tissues and normal testicular tissues were assessed by immunohistochemistry. 5-hmC staining is estimated as a percentage under every nuclear staining intensity score (0-3), 0 or 1 of which were regarded as 5-hmC reduction. The quantification of 5-hmC reduction is defined as the percentage of cells with 5-hmC staining scores of 0 and 1. According 5-hmC reduction of 80%, a 5-hmC reduction of <80% is regarded as "5-hmC high-level group", and a 5-hmC reduction of ≥80% is regarded as "5-hmC low-level group". Furthermore, Cox regression model was used to evaluate the prognostic value of all covariates.

**Results:** The median percentage of 5-hmC reduction in the PT-DLBCL group was 77.50% (60%-90%), the median 5-hmC reduction in the normal testicular tissues was 30% (20%-50%). Compared with normal testicular tissue, 5-hmC levels in PT-DLBCL tissue were significantly decreased (p<0.05). Of the 34 PT-DLBCL patients, 17 had tumors with relatively low 5-hmC expression (5-hmC reduction of ≥80%) and 17 had tumors with relatively high 5-hmC expression (5-hmC reduction of < 80%). 5-hmC expression was negatively correlated with international prognostic index (p = 0.037), while there was no significant difference in 5-hmC decrease among different groups of age at diagnosis, lactate dehydrogenase, testicular lymphoma involvement (unilateral or bilateral), Ki-67 and tumor diameter. Relatively low 5-hmC expression indicated shorter overall survival (OS) (5-year OS 50.2% vs 81.3%, p=0.022) and progression-free survival (PFS) (5-year PFS 38.5% vs 70.7%, p=0.001). Cox multivariate analysis of IPI (2-3 vs. 0-1), intrathecal prophylaxis (No vs. Yes), and 5-hmC reduction (≥80% vs. <80%) showed that 5-hmC reduction ≥80% (hazard ratio: 7.252, p = 0.005) and not receiving intrathecal prophylaxis (hazard ratio: 7.207, p =0.001) are independent risk factors for poor prognosis of PT-DLBCL.

**Conclusion:** Our results suggested that 5-hmC decline can be identified as a poor prognostic predictor for PT-DLBCL. It is necessary to further explore the underlying mechanism of this epigenetic marker to identify methods to re-establish 5-hmC levels and provide new targets for cancer therapy.

## Introduction

Primary testicular diffuse large B-cell lymphoma (PT-DLBCL) is a rare but highly aggressive extranodal lymphoma that accounts for approximately 1-9% of testicular malignancies and 1-2% of non-Hodgkin lymphoma^1^-^3^. PT-DLBCL mainly affects elderly men with a median age of 66-69 years. Although it is mainly limited to IE and IIE stages, PT-DLBCL has significant extranodal tropism, and its recurrence often involves the central nervous system (CNS), lung, adrenal glands, and bone marrow 4. In addition, PT-DLBCL responds poorly to the current empirical treatment regimen and has inferior outcome with no plateau in progression-free survival (PFS) and overall survival (OS) curves in historically retrospective studies compared with nodal DLBCL^4^. Moreover, there is still unmet medical need for patients who relapse. Due to the significant clinical and biological heterogeneity of PT-DLBCL^4^, the indicators for risk stratification have not yet met clinical needs. For instance, while the International Prognostic Index (IPI) has been frequently reported as a prognostic factor for DLBCL, for the majority of patients with PT-DLBCL who present with limited-stage disease, the IPI is typically <2 and therefore has limited prognostic utility^5^. PET-CT is the main criterion for staging and response evaluation, but it has shortcomings such as high false positive rate, high cost, radiation exposure, and inability to reflect the pathological characteristics and genetic information of the tumor. It is not suitable for developing targeted therapy and tracking clone evolution. Studies revealed that treatment enhancement measures based on PET-CT have not improved the prognosis^6^,^7^. Moreover, although rituximab had been reported to improve survival of PT-DLBCL in several retrospective studies^8^, it has not been observed in other studies^2^. Therefore, it is necessary to further explore risk stratification indicators to more accurately evaluate prognosis of PT-DLBCL patients, so as to screen high-risk patients and formulate individualized therapeutic regimens.

Epigenetic modifications play a vital role in biological and pathological processes through regulation of gene expression and genome stability without alterations in the DNA sequence. As an important epigenetic modification, the dynamic balance of DNA methylation and demethylation plays a significant role in the development and differentiation of mammals. Under the catalysis of Ten-eleven-translocation (TET) protein family, 5-methylcytosine (5-mC) is converted into 5-hydroxymethylcytosine (5-hmC), 5-formylcytosine (5-fC), and 5-carboxylcytosine (5-caC) through three consecutive oxidation reactions. Increasing studies revealed that 5-hmC is not only an intermediate product of DNA demethylation, but also a stable and active epigenetic marker^9^. The 5-hmC map drawn by whole-genome sequencing of various tissues supports its role as a gene expression marker^10^-^12^. 5-hmC is enriched in specific functional regions of the genome, especially enhancers, promoters and gene bodies^13^,^14^, suggesting that 5-hmC has a direct role in transcriptional regulation. In addition, many studies showed that the 5-hmC level of the protein-coding gene correlates positively with the mRNA expression intensity^14^,^15^. Moreover, researches of hepatocellular carcinoma, renal cell carcinoma, head and neck cancer, and melanoma have also demonstrated that 5-hmC decline in tumors is an independent prognostic factor of tumors and is closely related to the occurrence, progression and outcome of tumors^16^-^19^. However, the researches of 5-hmC in lymphoma are still in its infancy^20^,^21^. Thus, the alterations and possible role of 5-hmC in PT-DLBCL deserve further exploration. Besides, immunohistochemistry (IHC) has been proven to be a reliable method for detecting the level of 5-hmC^21^, and it can be used to detect the relative expression level of 5-hmC in PT-DLBCL tissues.

In this study, we used IHC to investigate the 5-hmC expression in normal testicular tissue and PT-DLBCL tissue, initially analyzed the correlation between the 5-hmC level and the clinical characteristics of patients with PT-DLBCL and its impact on the prognosis, providing experimental basis for exploring new biomarkers of PT-DLBCL.

## Materials and methods

### Study patients and data collection

We included 43 patients with PT-DLBCL treated in the Department of Hematology, Peking university first hospital from August 2000 to August 2020, thirty-four patients were confirmed mainly testicular involvement (stage I and II) by imaging (CT, PET-CT, ultrasound etc.) and pathology and were included in the subsequent analyses. All cases were reviewed by two hematopathologists, DLBCL was diagnosed according to the 2016 revision of the World Health Organization classification of lymphoid neoplasms^22^. The exclusion criteria were as follows: (i) in addition to involvement of the testicular, other extranodal sites were involved; (ii) any history of other types of tumors in the testis; (iii) any history of immunotherapy, radiotherapy and/or chemotherapy before diagnosis; (iv) no tissue specimens available; and (iv) most clinical data were not available. The 7 normal testicular tissues were obtained from prostate cancer patients who underwent bilateral orchiectomy for androgen deprivation therapy, of which no tumor involvement was confirmed by pathologists.

The clinical characteristics collected in this study included age at diagnosis, clinical manifestations (such as, the time from onset of symptoms to diagnosis, the predominant symptoms), testicular lymphoma involvement (unilateral or bilateral), tumor size, Lugano stage, Eastern Cooperative Oncology organization (ECOG) performance status score, lactate dehydrogenase (LDH), B symptoms, IPI, Ki-67, intrathecal prophylaxis, therapeutic regimens and survival status. Patients were all treated with the CHOP regimen (cyclophosphamide, doxorubicin, vincristine, and prednisone) or CHOP-like regimens with or without rituximab. OS was defined as the time from diagnosis to all-cause death or the last follow-up. PFS was defined as the time from diagnosis to progression, all-cause death or the last follow-up. Patients were followed up every three months for the first three years after chemotherapy, every six months in the fourth and fifth years, and then every year. The last follow-up date was December, 2020.

This study was part of a retrospective study (registration number: NCT03313271) which was approved by the ethical review board (approval number: 20171304) and have therefore been performed in accordance with the ethical standards laid down in the 1964 Declaration of Helsinki. Because the study is retrospective, the written informed consent is exempted.

### Immunohistochemical staining

A part of the surgical tissue specimen was fixed with 10% formalin at room temperature for 24 hours and embedded in paraffin. IHC analysis was performed on 4-μm thick sections. In short, after a series of standard procedures (deparaffinization, antigen retrieval, blocking endogenous peroxidase, blocking), sections were incubated with primary antibodies against 5-hmC (1: 1000, Abcam, Cat#: ab214728) overnight. This incubation was followed by incubation with peroxidase-conjugated anti-rabbit immunoglobulin G (Cat. No. GB23303, Servicebio, 1:200) at room temperature for 50 minutes. Then, in accordance with the protocol of the DAB chromogenic kit (Servicebio; Cat#: G1211), the slides were placed in phosphate buffer saline (PH 7.4) and washed 3 times with shaking on a decolorizing shaker, each time for 5 minutes. After the sections were dried slightly, freshly prepared DAB color developing solution was added to the circle, and the color development time was controlled under the microscope. The positive color was brownish yellow. The sections were washed with tap water to stop the color development.

### Scoring system

The intensity of 5-hmC nuclear staining was assessed by two experienced pathologists in a blind way. Scoring criteria: (1) All samples were assessed according to the 5-hmC nuclear staining intensity score (0-3): 0 points for light blue nuclear staining, 1 point for light brown nuclear staining, 2 points for mild brown nuclear staining, and 3 points for dark brown nuclear staining. (2) The percentage of every staining score in a high-power field was estimated. (3) Five high-power fields were randomly selected and the average value under every staining score was calculated for every tissue section. The average percentage of every staining score was calculated for every patient. (4) 5-hmC nuclear staining intensity scores of 0 or 1 were regarded as considerable 5-hmC reduction^21^. The quantification of 5-hmC reduction is defined as the percentage of cells with 5-hmC staining scores of 0 and 1. (5) According to the literature^21^, a 5-hmC reduction of 80% is selected as the threshold, that is, a 5-hmC reduction of <80% is regarded as "5-hmC high-level group", and a 5-hmC reduction of ≥80% is regarded as "5-hmC low-level group".

### Statistical analysis

The Kolmogorov-Smirnov method was used to determine whether the data conformed to the normal distribution. If continuous measures are normally distributed, two-sample t-test were conducted to compare the difference between 2 groups, otherwise the Mann-Whitney U test. Frequencies and percentages were counted for clinical parameters in different 5-hmC level groups and tested for differences by Chi-square and Fisher's exact tests. OS and PFS between the subgroups were estimated using Kaplan-Meier method and compared by the log-rank test. Univariate and multivariate cox-regression analyses were performed to determine independent prognostic factors of PT-DLBCL. All statistical analyses were conducted by IBM SPSS Statistics 22.0 software or GraphPad Prism 9. Differences were considered statistically significant at P < 0.05.

## Results

### Clinical Characteristics

A total of 34 patients with PT-DLBCL were included in this study, with a median age of 66 years (range 30-86) at diagnosis, and the median time from onset of symptoms to diagnosis in our hospital was 3 months (range 0.5-48). They mainly presented with testicular enlargement, hardness, and swelling, 26.5% of whom suffered from testicular pain or tenderness at the same time. Of the 34 patients with PT-DLBCL, 17.6% of patients had bilateral testicular lymphoma involvement. 26.5% of patients had B symptoms. All 34 patients received CHOP- or CHOP-like regimen, 5 without rituximab and 29 in combination with rituximab. The clinical characteristics of 34 PT-DLBCL patients are shown in Table 1.

### The 5-hmC level in PT-DLBCL tissue and normal testicular tissue

**Figure 1:**
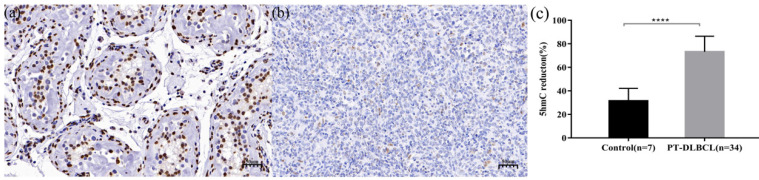
Immunohistochemistry (IHC) staining of 5-hmC in normal testicular. tissue (a) and PT-DLBCL(b). (c) 5-hmC expression was significantly lower in PT-DLBCL than normal testicular tissue by IHC. *** P < 0.0001.

Global 5-hmC levels were evaluated by IHC in normal testicular tissues (n = 7) and PT-DLBCL (n = 34) tissues. In normal testicular tissue, the morphology of seminiferous tubules was normal, and most of the nuclei staining were mild brown or dark brown nuclei, which were equivalent to medium positive and strong positive staining, respectively (Figure 1a). The median percent of 5-hmC reduction in 7 normal testicular tissue was 30% (range 20%-50%). In PT-DLBCL tissues, the structure of seminiferous tubules disappeared, medium-large atypical lymphocytes were diffusely proliferated and infiltrated, and most of the nuclei staining were light blue or light brown nuclear staining, which was negative or weakly positive (Figure 1b). The median percentage of 5-hmC reduction in 34 PT-DLBCL tissue was 77.50% (range 60%-90%). Compared with the 5-hmC reduction in normal testicular tissues, the reduction of 5-hmC in PT-DLBCL tissue was significant, and the difference was statistically significant by Mann-Whitney U test (P<0.0001, Figure 1c).

### Differences in clinical characteristics between two 5-hmC subgroups of patients

**Figure 2:**
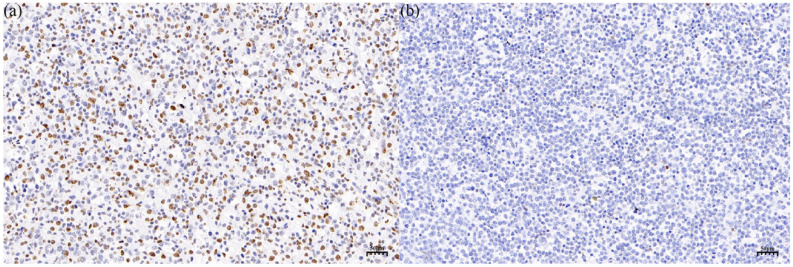
IHC staining of 5-hmC in the 5-hmC high-level group (a) and the 5-hmC low-level group (b), which respectively represented to a 5-hmC reduction of <80% and a 5-hmC reduction of ≥80% in PT-DLBCL**.**

According to the literature^21^, a 5-hmC reduction of 80% is selected as the threshold, 34 patients with PT-DLBCL were divided into 5-hmC high-level group ('5-hmC reduction <80%') and 5-hmC low-level group ('5-hmC reduction ≥80%') (Figure 2a and b). Among the 34 patients, there were 17 patients in the 5-hmC high-level group and 17 patients in the 5-hmC low-level group. To explore the potential value of 5-hmC in PT-DLBCL, we further investigated the relationship between clinical parameter stratification and 5-hmC level (Table 2). Remarkably, among the PT-DLBCL patients, the relatively low-level group was more likely to have a higher IPI score (≥2) compared with the 5-hmC high-level group of PT-DLBCL patients (p = 0.037). However, no significant correlations were noted between the 5-hmC level and age (>60/≤60 years) at diagnosis, tumor size (≥7.5cm/<7.5cm), LDH (normal/abnormal), testicular lymphoma involvement (bilateral/unilateral) or Ki-67 (≥90%/<90%) (p>0.05). In addition, there was no significant difference in chemotherapy regimen (P=0.650) as well as intrathecal prophylaxis (P=0.438) between the 5-hmC high-level group and the 5-hmC low-level group.

### The influence of 5-hmC level on patient survival

**Figure 3:**
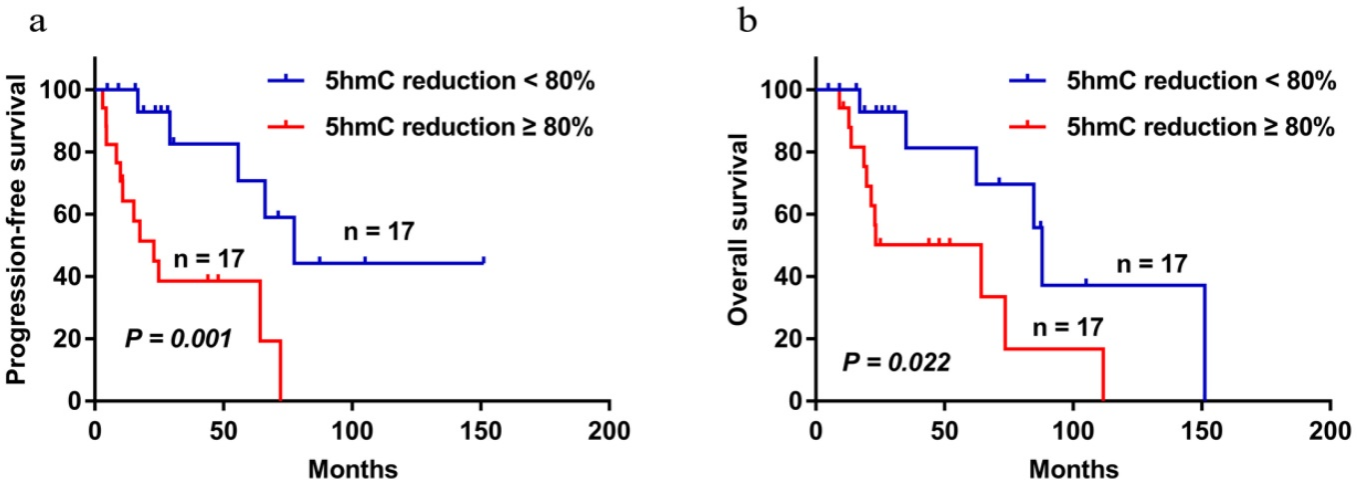
Progression-free survival (a) and overall survival (b) of PT-DLBCL patients with high 5-hmC staining and low 5-hmC staining by using the Kaplan-Meier method. *P* < 0.05 by log-rank test.

The median follow-up time of the 34 patients were followed up with a median follow-up time of 47.93 months (range 4.83-151.20). During the follow-up period, 17 patients died. The 5-year PFS and OS were 52.7% and 64.8%, respectively. Patients with low 5-hmC levels had worse 5-year PFS and OS compared with those who had high 5-hmC levels (5-year PFS 38.5% vs 70.7%,* P* = 0.001; 5-year OS 50.2% vs 81.3%, *P* =0.022) (Figure 3a and b). This finding indicates that low 5-hmC levels correlated with a poor prognosis among patients with PT-DLBCL.

### Univariate and multivariate Cox regression analyses for clinical features

Univariate and multivariate Cox-regression analyses were performed to determine independent prognostic factors of PT-DLBCL. Compared with those who had IPI (<2), intrathecal prophylaxis, and 5-hmC reduction <80%, patients with IPI (≥2), no intrathecal prophylaxis, or 5-hmC reduction ≥80% had worse 5-year OS by Cox univariate analysis (*P* < 0.05), while there was no significant difference on OS among different groups of age at diagnosis (> 60 vs. ≤ 60, *P* = 0.215), LDH (normal/abnormal, *P* = 0.619), testicular lymphoma involvement (unilateral or bilateral, *P* = 0.121), Ki-67(≥ 90% vs. < 90%, *P* = 0.665) and tumor diameter((≥7.5 vs. <7.5cm, *P* =0.930). A further Cox multivariate analysis of IPI (2-3 vs. 0-1), intrathecal prophylaxis (No vs. Yes), and 5-hmC reduction (≥80% vs.<80%) demonstrated that 5-hmC reduction ≥80% (HR: 7.252, 95% CI: 1.835-28.661; p=0.005) and no intrathecal prophylaxis (HR: 7.207, 95% CI: 2.230-23.296; p=0.001) were independent prognostic factor correlating with a shorter OS (Table 3). The higher the 5-hmC level, the better the prognosis. In addition, PT-DLBCL patients could benefit from intrathecal prophylaxis, achieving a better OS.

## Discussion

PT-DLBCL is a highly aggressive extranodal lymphoma. Compared with those with nodal DLBCL, patients with PT-DLBCL have shorter PFS and OS due to significant extranodal tropism and lack standard treatment regimens after relapse^4^. However, due to the significant clinical and molecular heterogeneity of PT-DLBCL, currently commonly used clinical risk stratification indicators still fail to meet the clinical needs of prognostic evaluation and monitoring of recurrence. Apparently, new insights into the unique pathophysiology of PT-DLBCL may facilitate significant clinical risk stratification indicators and novel therapeutic strategies. For example, a greater understanding of the pathophysiologic processes of PT-DLBCL shows that 60% to 96% of cases were activated B-cell-like (ABC) subtype in cell of origin^23^,^24^, the adverse prognosis by which may be attributed to chronic active B-cell receptor signaling and constitutive NF-κB and PI3K activation^25^. Analyses of genome in ABC subtype DLBCL have developed novel therapeutic strategies, such as ibrutinib (the inhibitor of Bruton's tyrosine kinase) and Pomalidomide (an immunomodulatory drug). There is evidence that both ibrutinib and Pomalidomide have excellent penetration of the blood-brain barrier and are well tolerated and effective^26^,^27^. Therefore, in the era of targeted therapy, biomarkers that reflect the genetic characteristics of tumors have the potential to evaluate the prognosis and be used for targeted drug development^28^. It is well known that the gene-specific and genomic changes of DNA methylation have been described in various subtypes of NHL^29^-^31^.Abnormal DNA methylation in B-cell lymphoma increases with the severity of the disease^32^. 5-hmC is not only an intermediate product of active DNA demethylation, but also proved to be a truly stable epigenetic marker. More and more studies have shown that changes in 5-hmC may promote the occurrence and progression of tumors, and are closely related to tumor staging, overall survival and recurrence^16^-^19^. This study aimed to explores the expression of 5-hmC in PT-DLBCL tissues and its influence on prognosis.

In this study, IHC showed that 5-hmC levels in PT-DLBCL were significantly reduced compared to those in normal tissues. This phenomenon has also been observed in other tumors. Qiu et al.^21^ performed 5-hmC staining by IHC in a large cohort of Mycosis fungoides patients (including those with patch stage, plaque stage, tumor stage and large cell transformed (LCT) MF) and benign inflammatory dermatoses (including lupus erythematosus, lichen planus, and psoriasis) and found that a progressive loss of 5-hmC was observed from patch/plaque stage to tumor stage and a remarkable loss of 5-hmC was observed in MF with LCT, which represents an aggressive state during MF progression. In addition, a decrease in 5-hmC has been observed in other solid tumors such as lung cancer, kidney cancer, and thyroid cancer^14^,^33^,^34^. Tian et al^35^ utilized the 5-hmC signature in circulating cell-free DNA of patients with esophageal cancer to construct a diagnostic model for predicting esophageal cancer, and the performance of predicting esophageal cancer reached a sensitivity of 93.75% and a specificity of 85.71%. The above indicate that 5-hmC reduction in PT-DLBCL compared with normal testicular tissue may be related to the development of PT-DLBCL, but more investigation is needed to identify the phenomenon and potential mechanisms. Various mechanisms have been proposed to explain tumor-related 5-hmC disorders, including gene mutation or down-regulation of ten-eleven translocation enzyme^36^, gene mutation of isocitrate dehydrogenase affecting the co-substrate of TET enzyme^14^, and potential abnormalities of regulatory factors^21^ so on. But the mechanism of 5-hmC alterations in different tumors may be different. In the future, experiments will be conducted to clarify the mechanism of 5-hmC dysregulation in PT-DLBCL.

Additionally, this study further investigated the relationship between clinical characteristics and 5-hmC level to explore the potential value of 5-hmC in PT-DLBCL. The results demonstrated that the 5-hmC relatively low-level group exhibited an increased risk of a higher IPI score (≥2) compared with the 5-hmC relatively high-level PT-DLBCL patients (p<0.05), indicating that 5-hmC decline has potential risk assessment capabilities. However, no significant correlations were noted between the 5-hmC level and Ki67, which may be explained by the generally high level of Ki67 in PT-DLBCL patients, with a median Ki67 of 90%. Furthermore, the Kaplan-Meier curve showed that a worse prognosis was associated with a lower 5-hmC level in PT-DLBCL (5-year PFS 38.5% vs 70.7%, p=0.001; 5-year OS 50.2% vs 81.3%, p=0.022). And 5-hmC was assessed as an independent prognostic factor for PT-DLBCL by univariate and multivariate Cox regression survival analyses. Increasing evidence shows that a reduction or even loss of 5-hmC is essential for various biological and pathological processes. For example, 5-hmC levels are almost universally depleted in Hodgkin lymphoma, and l-ascorbic acid can restore 5-hmC levels and induce tumor cell apoptosis in Hodgkin lymphoma cell lines^37^. Moreover, 5-hmC exhibits diagnostic and prognostic significance in percutaneous T-cell lymphoma 21. Studies of liver cancer, kidney cancer, head and neck cancer, and melanoma have also revealed that 5-hmC decline in tumor tissue is an independent prognostic factor of tumors and is closely related to tumor staging and overall survival^16^-^19^. And the treatment effect and recurrence status can be accurately tracked according to the change of the 5-hmC level on the identified genes^33^. Therefore, 5-hmC can be identified as a significant indicator to evaluate prognosis of PT-DLBCL. It is necessary to further examine the underlying mechanism of this epigenetic marker to identify a method to re-establish 5-hmC levels and provide new directions for cancer prevention and treatment.

Of course, this study has the limitation of only a few samples. However, considering the rarity of PT-DLBCL, this study still provides interesting novel information. More research, especially at the molecular level, needs to be conducted to explore the carcinogenic mechanism of 5-hmC.

## Conclusion

According to our results, 5-hmC levels are significantly decreased in PT-DLBCL and can be identified as a valuable prognostic predictor of PT-DLBCL. It is necessary to further explore the underlying mechanism of this epigenetic marker to identify methods to re-establish 5-hmC levels and provide new targets for cancer treatment.

## Figures and Tables

**Table 1:** Clinical Characteristics of 34 Patients with PT-DLBCL

Characteristics	group	n (%)
Age		
	≤60	10(29.4%)
	>60	24(70.6%)
Testicular lymphoma involvement		
	Unilateral	28(82.4%)
	Bilateral	6(17.6%)
Tumor size (cm)		
	< 7.5	24(70.6%)
	≥ 7.5	10(29.4%)
LDH		
	Normal	24(70.6%)
	Abnormal	10(29.4%)
IPI		
	0-1	19(55.9%)
	2	10(29.4%)
	3	5(14.7%)
Ki-67(%)		
	<90	14(41.2%)
	≥90	20(58.8%)
Intrathecal prophylaxis		
	Yes	24(70.6%)
	No	9(26.5%)
	Missing	1(2.9%)
Chemotherapy regimen		
	CHOP (like)	5(14.7%)
	R+CHOP (like)	29(85.3%)

Abbreviations: LDH, Lactate dehydrogenase; IPI, International Prognostic Index; CHOP, cyclophosphamide, doxorubicin, vincristine, and prednisone; R, rituximab.

**Table 2:** Correlation analysis by Chi-square between 5-hmC levels and clinical parameter stratification in PT-DLBCL patients.

Characteristics	5-hmC expression		p
	High (n)	Low (n)	
Age			*P* =1.000
≤60	5	5	
>60	12	12	
Testicular lymphoma involvement			*P* =0.175
Unilateral	16	12	
Bilateral	1	5	
Tumor size (cm)			*P* =0.259
≤7.5	14	10	
>7.5	3	7	
IPI			*P* =0.037
0-1	13	6	
2-3	4	11	
Ki-67			*P* =0.728
<90%	8	6	
≥90%	9	11	
LDH			*P* =0.057
Normal	15	9	
Abnormal	2	8	
Chemotherapy regimen			*P* =1.000
R+CHOP (like)	15	14	
CHOP (like)	2	3	
Intrathecal prophylaxis			*P* =0.438
Yes	13	11	
No	3	6	
Missing	1	0	

Abbreviations: LDH, Lactate dehydrogenase; IPI, International Prognostic Index; CHOP, cyclophosphamide, doxorubicin, vincristine, and prednisone; R, rituximab.

**Table 3:** Univariate and multivariate Cox regression analyses for overall survival among 34 PT-DLBCL patients.

PT-DLBCL patients	Univariate analysis			Multivariate analysis		
	HR	95%CI	p-value	HR	95% CI	p-value
Age at diagnosis (> 60 vs. ≤ 60)	2.244	0.625-8.051	0.215	-	-	-
LDH (abnormal vs. normal)	1.298	0.464-3.630	0.619	-	-	-
Ki67 (≥ 90% vs. < 90%)	1.253	0.452-3.474	0.665	-	-	-
IPI (2-3 vs. 0-1)	5.021	1.411-17.863	0.013	-	-	0.619
Testicular lymphoma involvement (bilateral vs unilateral)	2.432	0.791-7.475	0.121	-	-	-
Tumor size (≥7.5 vs. <7.5cm)	0.955	0.344-2.651	0.930	-	-	-
5-hmC reduction (≥80% vs.<80%)	3.336	1.125-9.896	0.030	7.252	1.835-28.661	0.005
Intrathecal prophylaxis: No vs. Yes	4.449	1.573-12.583	0.005	7.207	2.230-23.296	0.001
